# Single-Item, Self-Rated Health is a Useful Indicator of Health in Myofascial Temporomandibular Disorders

**DOI:** 10.11607/ofph.2045

**Published:** 2018-10-26

**Authors:** Vivian Santiago, Karen Raphael

**Affiliations:** Department of Oral and Maxillofacial Pathology, Radiology and Medicine New York University College of Dentistry New York, New York, USA; Department of Oral and Maxillofacial Pathology, Radiology and Medicine New York University College of Dentistry New York, New York, USA

**Keywords:** general health, pain, self-perceived health, self-rated health, TMD

## Abstract

**Aims:**

To *(1)* examine differences in self-rated health (SRH) between a group of women with myofascial temporomandibular disorders (mTMD) and controls; *(2)* determine the extent to which pain, mental health, and physical function mediate these differences; and *(3)* explore specific mTMD symptoms and impairments explaining SRH among mTMD cases.

**Methods:**

An existing dataset of a sample of women with mTMD (n = 125) and a group of demographically similar controls (n = 49) was used. SRH was measured via a single item with 5 answer options ranging from poor (SRH = 1) to excellent (SRH = 5). Bodily pain, mental health, and physical function were measured with the Short-Form Health Survey. Regression analyses with SRH as the outcome were conducted.

**Results:**

mTMD cases reported poorer SRH compared to controls, and bodily pain score fully mediated these lower scores. Physical function partially mediated the association between mTMD and SRH, while mental health did not explain much of the variance in SRH. This pattern held in case-only analyses. The association was not explained by mTMD-specific symptoms or by localized mTMD pain severity, although mTMD disability was independently associated with lower SRH.

**Conclusion:**

SRH is a simple and useful tool to consider in mTMD research, as it discriminates between cases and controls based on pain and physical function and is associated with mTMD disability.

Self-rated health (SRH), or general health status, is a powerful predictor of morbidity^1,2^ and mortality^1,3^ across public health research in a variety of populations, even after multiple demographic and clinical indicator adjustments, making it a uniquely useful health indicator. Despite this powerful evidence, exploration of single-item measures of SRH in chronic pain research has been limited. Given their significant predictive power in research on other health outcomes (such as cardiovascular disease^4^ and all-cause mortality^2,3^), SRH presents potential utility for research on chronic pain conditions like temporomandibular disorders (TMD), which are plagued with the challenges inherent in measuring and understanding the pain experience. In chronic pain research and treatment, the main outcomes of interest are often pain measures themselves; ie, pain intensity at the clinical location of concern or other assessments explicitly anchored to the experience of pain, such as functional outcomes.^5,6^ Both of these measures (pain intensity and functional outcomes), although critical, cannot be easily obtained from disorder-free individuals for comparison or from the general population, where many individuals may not be experiencing pain. Moreover, these outcomes are inherently similar to the disorder under study—chronic pain—because they are anchored to pain, creating a circularity of measurements in the outcomes studied that may limit interpretation. Since SRH is a measure that can be easily obtained, has been well validated, and can be used throughout the stages of illness, if applicable to TMD, it can provide information with adequate time to intervene and improve outcomes. Therefore, SRH as an additional outcome measure in orofacial pain research would have the added advantage over typical measures of pain and function of not being tautologically used to also define the presence of the health condition characterized by chronic pain itself.

Despite some research on its applicability in chronic pain samples^7–16^ (including TMD^17,18^), few studies focus on SRH, systematically examine differences in SRH between chronic pain patients and controls, and explore factors mediating this association. Therefore, despite the potential use of SRH, clinicians and researchers need more information to identify clearer ways of intervening in the management of TMD (or chronic) pain to improve SRH. By identifying the mediating factors that drive TMD patients’ ratings of their overall health, including the role of TMD-related symptoms, measures of SRH can be better utilized in research on chronic pain and treatment-related improvements. To the authors’ knowledge, no study has examined this in myofascial TMD (mTMD). Consequently, the aims of this study were to: *(1)* examine differences in a single-item measure of SRH between women with mTMD and controls; *(2)* confirm that these SRH differences are at least in part mediated by true health effects via some of the specific health domains relevant in the study of chronic pain^5^ (ie, bodily pain, mental health, and/or physical function); and *(3)* explore specific mTMD symptoms and impairments predicting SRH among women with mTMD.

## Materials and Methods

### Participants

Data were drawn from an existing dataset of a case-control study^19^ with data on the measures of interest in 125 women with mTMD and a group of demographically similar controls (n = 49).^19^ In the original study, cases were recruited from the Facial Pain Clinic at New York University (NYU) College of Dentistry and via advertisements at the university clinics. Controls were also recruited from NYU dental clinics and by referral from participating cases and were selected to match the demographic composition of the case group. All participants underwent a full informed consent process before enrollment, and the study received all necessary approvals through the NYU School of Medicine Institutional Review Board (IRB#07-303). Participants’ sociodemographic and clinical characteristics are summarized in Table 1.

### Measures

#### Clinical Research Examinations.

mTMD was assessed using the Research Diagnostic Criteria for TMD (RDC/TMD) to diagnose Group I myofascial pain based on Axis I criteria^20^ (including palpation) and clinical judgment that the pain was primarily muscular rather than joint based. As such, a full RDC/TMD examination, including classification of other possible TMD diagnostic groups, was not administered. To ensure bodily pain ratings (see below) among cases were not due to a widespread pain condition such as fibromyalgia, fibromyalgia was explored as a covariate in case-only analyses. Fibromyalgia was diagnosed using the American College of Rheumatology (ACR) 1990 criteria; ie, clinical research examination of 11 or more tender points to palpation and report of four-quadrant pain.^21^

#### Interview-Based Measures.

Outside of the clinical research examination, study personnel collected participant information via patient interviews. The RDC/TMD Patient History Questionnaire^20^ was used to assess facial pain intensity, jaw-related symptoms, specific activities impaired by facial pain, and facial pain interference with daily activities.^20^ Facial pain–related interference items were summarized into total disability points as described in the RDC/TMD Axis II instructions for graded chronic pain.^20^ Total disability points represent the summary of three questions on facial pain interference with daily activities and the number of days kept from daily activities due to facial pain. Responses were positively skewed and were therefore further dichotomized as TMD disability (1) if patients had any points vs no disability (0) if patients had no points.

SRH was also measured using the RDC/TMD Patient History Questionnaire item 1,^20^ which asks patients to rate their general health on a scale from 1 to 5, with answer options ranging from excellent to poor. These answers were reverse coded as 1 = poor and 5 = excellent.

To examine common health domains across cases and controls, subscales from the widely used Short Form Health Survey (SF-36)^22,23^ were employed because the SF-36 is validated across populations and therefore applicable to both cases and controls. Specifically, the bodily pain score, which includes both pain severity and pain interference with normal work in the past 4 weeks, was used along with the physical function and mental health domains because these are three related but distinct health domains of the SF-36, potentially applicable across cases and controls. SF-36 scores used for the bodily pain, physical function, and mental health domains were age-standardized *z* scores (range −1 to 1) based on the US population of women, where higher positive values represent less pain, better physical functioning, and better general mental health.

### Statistical Analyses

Descriptive and linear regression analyses with SRH as the dependent variable were conducted using Stata/SE.^24^ First, to determine whether mTMD status significantly predicted lower SRH, the full sample of cases and controls was analyzed, with mTMD case status as a predictor. Sociodemographic variables were explored as confounders. Second, to understand what areas of health may be mediating the hypothesized association, the Baron and Kenny approach to mediation using linear regression models was used.^25^ After establishing that each explored SF-36 domain was associated with mTMD and SRH and that there was no SF-36 domain*mTMD interaction, the three SF-36 domains were separately added to the above model with mTMD predicting SRH. To determine if a given SF-36 domain mediated potential differences in SRH between cases and controls (ie, mediated the association between mTMD and SRH), the beta coefficients for mTMD status in the unadjusted and mediator-adjusted models were compared for change. A decrease in the magnitude of effect (ie, regression coefficient) of the mTMD-SRH association after inclusion of the given SF-36 domain indicates that the domain either fully or partially mediates the association. Separately, to confirm the conclusions, the analyses were repeated using the paramed function in Stata,^26^ a recent statistical advancement that allows the multiple procedures presented here in separate models to be run simultaneously to assess the direct and indirect effects of a mediator in the association between a predictor and an outcome (data not shown).

Once the above analyses provided clues to factors mediating the association between mTMD and SRH, the next statistical tests were designed to examine how mTMD-specific symptoms may be playing a role in SRH. These analyses were conducted using only the cases because full data on mTMD symptoms were only systematically assessed among individuals who met the diagnostic criteria for mTMD. Symptoms were explored individually and in summary (ie, the total number of jaw-related symptoms and total number of activities affected by facial pain). A series of regression models were run among cases only. First, to confirm that the analyses of SF-36 domains predicting SRH conducted in the full sample (including the controls) held among the cases, the SF-36 domains were examined among cases only. Then, a series of models determined whether localized or widespread pain (ie, comorbid fibromyalgia) best explained SRH among the cases. Separately, a series of models examined which mTMD symptoms and related impairments predicted SRH. Finally, combining information from conclusions across analyses, one final model was fitted with the most predictive variables observed in the previous regressions. A backward selection regression procedure where *P* < .05 was considered significant using a full model with all the identified predictors was used to determine which of these variables (across the SF-36 domains, pain location, and mTMD-related symptoms and disability) best predicted SRH among the cases.

## Results

### Differences in SRH Between mTMD Cases and Controls

Participant sociodemographics and clinical characteristics are summarized in Table 1. Women in the mTMD group reported lower SRH compared to controls; the mean score among cases was about a half point lower than controls. As expected, none of the examined sociodemographic variables were associated with case status, since cases and controls were originally matched on demographics. Therefore, none were included as possible confounders in the multivariate models.

### SF-36 Domains Mediating Case-Control Differences in SRH

The three domains of the SF-36 were significantly correlated with each other. Bodily pain and physical function had a moderate correlation (*r* = 0.57, *P* < .05), but each was only weakly associated with mental health (*r* = 0.36, *r* = 0.28, respectively; *P* < .05 for both). Results examining which of the SF-36 domains mediated these lower health ratings among cases compared to controls are summarized in Table 2. All three domain mean *z* scores were significantly lower in the cases compared to controls and were significantly associated with SRH. However, bodily pain and physical function explained a greater portion of SRH variance than did mental health. As is shown in Table 2, once bodily pain was introduced into the model of mTMD predicting SRH, the mTMD-SRH association was no longer significant, with a coefficient much closer to the null value of 0. This suggests that bodily pain fully mediates the association between mTMD and SRH. In a separate model, physical function only partially mediated this association, as the mTMD-SRH coefficient was only partially reduced from −0.50 to −0.29 and remained significant. This mediation is potentially the result of the correlation between bodily pain and physical function. The impact of mental health on the TMD-SRH association was minimal, as it explained very little variance in SRH and, when entered into the TMD-SRH model, only slightly lowered the coefficient to −0.41. The paramed procedure^26^ confirmed these conclusions.

### Predictors of SRH Among mTMD Cases

Predictors of SRH among cases in unadjusted simple linear regression models are summarized in Table 3. Similar results were found in the case-only analyses for bodily pain and physical function as predictors of SRH (Table 4, Model 2), but mental health did not reach significance. Facial pain intensity measured in various ways predicted SRH, explaining a small proportion of variance. Given the impact of bodily pain on the difference in SRH between cases and controls, it was decided to test whether SRH among cases was better explained by localized or widespread pain. To do so, the facial pain intensity measure that was most predictive in the multivariate model—worst pain in the last month—was used as a measure of localized pain, and a positive research diagnosis for fibromyalgia was used as the indicator of widespread pain. Although both were significant and independent predictors of SRH in the multivariate model (Table 4, Model 2), in the sample selected for facial pain, it was surprising that the localized pain intensity measure did not appear to explain a greater proportion of variance than the presence of comorbid fibromyalgia (Table 3). Although there was no comparable pain intensity measure in the last 6 months for widespread pain, a bodily pain intensity measure is included as part of the bodily pain scale of the SF-36 referencing the past 4 weeks. As noted below, localized pain intensity fell out of the model with the addition of the bodily pain score. For comparability, one of the items from the bodily pain scale that measures severity of bodily pain in the past 4 weeks (none to very severe) was substituted for fibromyalgia in the above analysis and obtained a similar result (data not shown).

### mTMD-Specific Symptoms and SRH Among mTMD Cases

**Fig 1.**
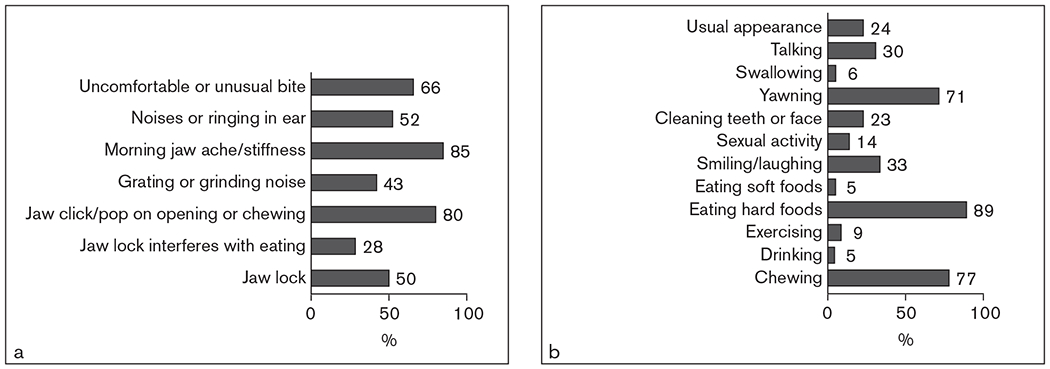
Percent of women with mTMD reporting mTMD-specific symptoms. **(a)** Symptoms related to jaw function. **(b)** Activities reported to be affected by facial pain.

How specific mTMD symptoms, activities affected by facial pain, and related impairments predicted SRH was also examined. The frequencies of mTMD symptoms are summarized in Fig 1. In a series of regressions, of the mTMD jaw-related symptoms, only uncomfortable or unusual bite significantly predicted SRH, and of the activities affected by facial pain, only sexual activity, talking, and having usual appearance predicted SRH (Table 3). The total number of activities affected was more predictive than any of the individual items. As such, the final model representing symptoms and impairments included only mTMD disability and the number of activities affected by facial pain (Table 4, Model 3).

### Best Predictors of SRH Among Women with mTMD Cases

The best predictors identified in each step described above were entered at once into the backwards selection model predicting SRH among cases to identify which were the best predictors in the multivariate model at the *P* < .05 level. The variables significantly predicting SRH and therefore retained in the model were bodily pain, physical function, fibromyalgia, and mTMD disability (Table 4, Model 4). Surprisingly, none of the localized symptoms, including facial pain intensity, added to the model above the predictive value of the other variables in explaining SRH. Fibromyalgia remained significant in this multivariate model, but so did bodily pain, suggesting that the bodily pain impact on SRH among women with mTMD is not merely due to comorbid fibromyalgia. The final model explained 35% of the variance in SRH.

## Discussion

Similar to research on other chronic conditions,^2^ including the limited available research on chronic pain^7^ and TMD,^17,18^ mTMD status was associated with significantly lower ratings of SRH in this sample of women. Given the limited research on mTMD and SRH, there are few studies with which to integrate these findings; therefore, in addition to single-item measures of SRH, studies that used SRH as part of multi-item self-reported general health status measures and studies of TMD generally (not just the myofascial subtype) are reviewed.

The Orofacial Pain Prospective Evaluation and Risk Assessment (OPPERA) study on first onset of TMD examined single-item SRH^18^ and found that poorer SRH predicted first onset of TMD after adjusting for study site and patient demographics.^18^ Another study on TMD examined SRH via the general health domain of the SF-36 in relation to duration of TMD and found that SRH was significantly lower compared to the general population regardless of level of duration (< 1 year, 1–3 years, or > 3 years) and that SRH differences by duration were not significant.^17^ These publications did not report specifically on the myofascial subtype.

In addition to its established predictive power on general morbidity^1^ and mortality,^3^ the broader chronic pain literature provides further evidence of the potential utility of SRH. SRH in chronic pain has been underexplored or is often not the primary aim; nonetheless, studies have used SRH in a variety of ways. SRH has been used in chronic pain samples to understand its impact on the onset and persistence of chronic pain, as in the above-cited TMD studies. Another prospective community study on chronic pain explored SRH as the general health domain of the SF-36 and found that SRH predicted onset and persistence of chronic pain (ie, failure to recover from pain) in adjusted models.^8^ Other longitudinal studies have found that lower SRH predicts later pain,^9^ but longitudinal studies, to the present authors’ knowledge, have not examined whether individuals with chronic pain have lower SRH over time. Nonetheless, an association between lower SRH and chronic pain has been found in both clinical^11^ and population samples^7^ and in both cross-sectional^10^ and longitudinal^8,9,18^ study designs.

SRH has also been used to examine the impact of comorbidities on health, including the added burden of chronic pain to existing chronic conditions,^12^ and to understand how different chronic pain conditions may impact health in different ways.^13,14^ Fibromyalgia is consistently associated with poorer ratings of health across domains across studies, including general health ratings using the SF-36 and its abbreviated version, the SF-12, even compared to other pain conditions.^27^ Few studies on fibromyalgia focus on single-item measures of SRH. One study used a question similar to that used in the present study and found that a group with fibromyalgia or chronic fatigue syndrome had nearly four times the odds of poor/fair health and over twice the odds after adjusting for multi-morbidity.^16^ Therefore, fibromyalgia appears to be a strong driver of poor SRH that should be accounted for when examining other chronic conditions.

Health services evaluation research has also employed SRH as an outcome using data from hospital electronic records to evaluate the costs and effectiveness of treatment modalities on integrative care.^28^ Intervention studies have also used SRH and global health measures to examine intervention-related improvements on health.^15^ Sundstrup et al found that exercise-based interventions improved SRH scores among workers suffering from lower back problems.^15^ Hence, the utility of SRH measures, including simple single-item measures^10^ for the study of chronic pain, have been put to good use by some, but there is room for improvement. Clinical trials are increasingly incorporating patient self-assessments of their perceived improvement using measures recommended by IMMPACT,^6^ like the Patient Global Impression of Change, because of their utility in helping to determine clinically important treatment-related improvements. Similarly, measures of overall health that are not anchored to pain or treatment and that can be collected repeatedly may be of particular utility when studying treatment efficacy and effectiveness in TMD and other chronic pain conditions. However, in order to do so, a body of evidence on SRH in TMD and in other chronic pain conditions is needed.

The present study begins to fill this gap by adding to the evidence on the utility of SRH for capturing mTMD-specific effects. As expected, differences between cases and controls were primarily mediated by the bodily pain and physical function SF-36 domains. These domains, along with mental health, are of particular interest in chronic pain research as clinically meaningful indicators of the condition’s progression and management.^6^ Among women with mTMD, self-reported localized mTMD symptoms—including localized pain intensity and mTMD disability—did predict SRH but did not fully explain the impact of bodily pain and physical function on SRH. This indicates that there is an aspect of the mTMD chronic pain experience that is not merely a sum of localized pain and mTMD symptoms. The whole of the chronic pain experience appears to be more than the sum of its parts. Nevertheless, after adjusting for these factors, having at least one TMD Axis II disability point lowered the mean SRH score by a third of a point.

Despite the existing evidence of the psychologic impacts of chronic pain and TMD specifically,^29^ the present results show that the association between SRH and mTMD is not primarily explained by the potential impact of psychologic distress as measured by the SF-36 mental health scale. A similar conclusion was reached in analyses not detailed here by substituting the Symptom Checklist-90-Revised (SCL-R-90)^30^ depression score for the SF-36 mental health domain. This supports the validity and robustness of SRH for capturing impressions of overall health rather than solely capturing the respondents’ current psychologic state.

Given these findings, SRH appears to be a useful measure that can augment pain intensity and function as potential outcomes in mTMD research and clinical practice. Since mTMD is a pain condition, pain intensity measures are critical in the process of diagnosis, so it is necessary but inherently tautological to also use these measures as primary outcomes, particularly if the best expected outcome is management of pain to improve overall health and wellbeing, not the total elimination of pain. Moreover, SRH can add to the literature on quality of life and functional outcomes with a simpler single-item measure that is even more universal in nature because it is applicable throughout the course of illness and across disorders, including co-occurring chronic pain conditions or even among control participants. It is a simple metric for use as a patient outcome worth monitoring and targeting for improvement, given its predictive power for morbidity and mortality risk. As a result, its use can connect research across conditions. Additionally, given its broad-based use in the medical and public health literatures, SRH can help connect mTMD to and inform the broader health literature.

## Limitations

Temporal ordering of the examined associations is a noteworthy limitation of this study. Although the time periods referenced in the diagnosis of mTMD implicitly ensure that mTMD predated the assessment of SRH and SF-36 domains, since assessments were otherwise concurrent, it cannot be ruled out that the direction of effect may not be as inferred. For example, the OPPERA study found baseline SRH to be predictive of TMD onset.^18^ In addition, they also found that baseline physical and mental component summaries of the SF-12 Health Survey (version 2) significantly predicted TMD onset. Given this existing evidence, it is unclear to what extent SRH is a result of TMD or associated with factors leading to its onset. However, the present study specifically screened for chronic TMD and included only women. Moreover, these results differed from OPPERA^18^ in that mental health did not explain much of the differences in SRH between cases and controls or within cases. Therefore, it is likely that the new TMD-onset findings in the mixed gender OPPERA sample may not apply to this chronic mTMD sample of women. Similarly, valid examination of mediation can only be done in a longitudinal time frame, and these were cross-sectional data with the temporal ordering issues noted above. Therefore, future studies should re-examine these associations in longitudinal designs.

Another potential limitation is that other comorbid conditions, including co-occurring chronic pain conditions, were not accounted for, except for fibromyalgia. However, an ACR-based research clinical examination for fibromyalgia was included, which is a prototypical chronic pain condition. Nevertheless, the role of fibromyalgia in explaining the lower mean SRH scores among cases compared to controls could not be explored because of the low prevalence of fibromyalgia among the controls (only one control participant met the criteria). Case-only analyses did include fibromyalgia status, but bodily pain also remained significant in the multivariate model (Table 4). In addition, treatments that are ubiquitous among mTMD women were not controlled for, including prevalent medication use primarily consisting of nonsteroidal anti-inflammatory drugs. However, the number of medications reported for facial pain was examined, but as this did not add significantly to the models, the data were not presented.

Finally, the present sample included only women. Although TMD prevalence is higher among women^31^ and women report poorer SRH compared to men in national surveys,^32^ most studies find that SRH has been found to be more predictive for the risk of mortality among men than women.^32^ Despite this, the OPPERA study found a significant association between SRH and onset of TMD in a sample including men, although results were not stratified by gender or specific to the myofascial subtype. Nonetheless, it is unclear how these findings would apply to men with mTMD.

## Conclusions

Women with mTMD report lower SRH than women without mTMD, and this is due primarily to bodily pain and partially to physical function likely related to pain. However, among women with mTMD, localized pain intensity and specific symptoms were not the primary drivers of lower SRH, further highlighting the need to consider mTMD as a chronic pain condition with impacts on health beyond what is explained by localized symptoms. Together, the authors hope these findings begin to identify ways clinicians and researchers can improve the overall health of women with TMD and better integrate TMD research into the broader public health literature on SRH.

## Figures and Tables

**Table 1:** Participant Sociodemographic and Clinical Characteristics

	Total sample		mTMD patients		Controls		
	N	Measure	n	Measure	n	Measure	*P* value
Age (y)							
Range	174	19–78	125	19–78	49	19–71	
Mean (SD)		39.2		40.1(1.3)		36.8 (2.0)	.171
Median		36.5		37		32	

Education, %	172		123		49		.605
High school or less	36	21	27	22	9	18	
> High school, < college	95	55	65	53	30	61	
≥ College	41	24	31	25	10	20	

Income, %	165		119		46		.985
< $15,000	49	30	35	29	14	30	
$15,000 to < $50,000	57	35	41	34	16	35	
≥ $50,000	59	36	43	36	16	35	

Hispanic, %	38	22	27	22	11	23	.933

Age of facial pain onset (y)	–	–	125		–	–	
Range	–	–		10–66	–	–	
Mean (SD)	–	–		29.5 (13.7)	–	–	
Median	–	–		25	–	–	

Years since facial pain onset			125				
Range	–	–		0.33–50	–	–	
Mean (SD)	–	–		10.6 (10.5)	–	–	
Median	–	–		7	–	–	

Worst pain intensity in last month (0–10 NRS)			125				
Mean (SD)	–	–		7.6 (2.1)	–	–	
Median	–	–		8	–	–	

Fibromyalgia,^a^ %	–	–	26	21	–	–	

Self-rated health							
Mean (SD)	174	3.6 (0.91)	125	3.5 (0.95)	49	4 (0.74)	.001

SF-36 domains, mean (SD)							
Bodily pain	173	−0.35 (1.04)	125	−0.74 (0.90)	48	0.66 (0.60)	.000
Physical function	173	0.20 (0.73)	125	0.09 (0.79)	48	0.48 (0.42)	.002
Mental health	173	−0.11 (1.02)	125	−0.28 (1.0)	48	0.35 (0.86)	.000

*P* values are provided for differences between cases and controls (chi-square test was used for differences in frequencies and *t* test for differences in means). SD = standard deviation; NRS = numeric rating scale; SF-36 = Short Form Health Survey (higher positive values represent less bodily pain, better physical functioning, and better general mental health).

a One control participant met criteria for fibromyalgia, but analyses including fibromyalgia are limited to the case-only analysis, so the data are not provided here.

**Table 2:** Results of Mediation Analyses^a^

Predictors	Simple linear regressions^b^			Multiple regression models adjusted for given SF-36 domain^c^								
				Bodily pain as mediator			Physical function as mediator			Mental health as mediator		
	Coef	95% CI	R^2^	Coef	95% CI	Adj R^2^	Coef	95% CI	Adj R^2^	Coef	95% CI	Adj R^2^
mTMD	−0.50	−0.80,−0.20	0.06	0.17	−0.17, 0.51	0.24	−0.29	−0.57,−0.01	0.23	−0.41	−0.72,−0.10	0.07

SF-36 domains												
Bodily pain	0.44	0.32, 0.56	0.25	0.48	0.34, 0.63							
Physical function	0.58	0.42, 0.75	0.22				0.54	0.37, 0.71		0.14	0.00, 0.28	
Mental health	0.19	0.06, 0.33	0.04									

a The dependent variable in all regression models is the self-rated health measure (SRH). All models included 173 observations due to 1 control participant not completing the SF-36.

b Simple linear regression models provide the unadjusted association between the given indicator (mTMD) and SRH, including the total effect of mTMD on SRH.

c Models adjusted for the given SF-36 domain test whether addition of that domain reduces the coefficient of the mTMD-SRH association. Partial reduction suggests partial mediation. If the mTMD-SRH association approaches the null value and is no longer significant, then the SF-36 domain fully mediates the mTMD-SRH association. Additional mediation analyses using the paramed procedure in Stata to test mediation separately for each SF-36 domain support the conclusion reached by comparison of coefficients across these models: Bodily pain fully mediates the association between mTMD and SRH, while physical function partially mediates the association. Mental health has little impact.

Coef = coefficient; CI = confidence interval; Adj = adjusted.

**Table 3:** Predictors of Self-Rated Health Among mTMD Cases: Unadjusted Simple Linear Regression Models

	Coef	*P* value	R^2^
Age	**−0.007**	**.163**	**0.02**

SF-36 domains			
Bodily pain	**0.51**	**.000**	**0.24**
Physical function	0.57	.000	0.22
Mental health	**0.11**	**.180**	**0.01**
Fibromyalgia (presence/absence)	**−1.02**	**.000**	**0.15**

Facial pain			
Current	**−0.08**	**.031**	**0.04**
Worst (last 6 mo)	**−0.15**	**.000**	**0.11**
Average (last 6 mo)	**−0.14**	**.002**	**0.07**
Characteristic pain intensity	**−0.17**	**.000**	**0.10**
Years since onset	−0.01	.171	0.02

Facial pain–related impairments			
TMD disability (any disability points vs none)	**−0.71**	**.000**	**0.11**

mTMD-related symptoms			
Jaw lock	0.09	.605	0.00
Jaw lock interferes with eating	−0.33	.079	0.02
Jaw click/pop on opening mouth/chewing	0.12	.573	0.00
Grating or grinding noise	−0.06	.735	0.00
Morning jaw ache/stiffness	−0.04	.880	0.00
Noises/ringing in ear	−0.30	.081	0.02
Bite feels uncomfortable or unusual	**−0.44**	**.014**	**0.05**
Total no. of mTMD-related symptoms	−0.07	.137	0.02

Activities affected by facial pain			
Chewing	−0.39	.054	0.03
Drinking	−0.52	.190	0.01
Exercising	−0.54	.069	0.03
Eating hard foods	−0.25	.362	0.01
Eating soft foods	−0.35	.385	0.01
Smiling/laughing	−0.27	.141	0.02
Sexual activity	**−0.64**	**.007**	**0.06**
Cleaning teeth or face	−0.20	.329	0.01
Yawning	−0.04	.813	0.00
Swallowing	−0.53	.155	0.02
Talking	**−0.52**	**.004**	**0.07**
Having your usual appearance	**−0.39**	**.049**	**0.03**
Total no. of activities affected by facial pain	**−0.15**	**.000**	**0.10**

Bold numbers represent statistically significant estimates at *P* < .05 in separate unadjusted simple linear regression models, each with only the noted predictor of SRH.

**Table 4:** Best Predictors of Self-Rated Health Among mTMD Cases by Predictor Group

	(Model 1) SF-36 domains (n = 125)			(Model 2) Localized vs wide spread pain (n = 124)			(Model 3) mTMD symptoms and disability (n = 122)			(Model 4) Best predictors (n = 121)		
	Coef	95% CI	Adj R^2^	Coef	95% CI	Adj R^2^	Coef	95% CI	Adj R^2^	Coef	95% CI	Adj R^2^
Bodily pain	0.34	0.14, 0.53	0.28							0.23	0.03, 0.43	0.35

Physical function	0.35	0.13, 0.57								0.31	0.09, 0.53	

Fibromyalgia				−0.79	−1.17,−0.41	0.21				−0.49	−0.86,−0.13	

Worst facial pain intensity (last 6 mo)				−0.12	−0.18,−0.04							

TMD disability							−0.49	−0.91,−0.07	0.12	−0.35	−0.69,−0.00	

No. of activities affected by facial pain							−0.10	−0.19,−0.01				

Each column represents a multiple regression model for the named group of variables that were significant in the unadjusted simple linear regressions (see Table 3) for the given domain and that remained significant predictors of SRH when entered into the multivariate model.

Model 4 represents all of the variables explored that remained significant predictors of SRH after a backwards selection regression procedure (*P* < .05). Mental health did not significantly predict SRH among cases; therefore, it is not included. Coef = coefficient; CI = confidence interval; Adj = adjusted.
